# The e-EPIDEMIOLOGY Mobile Phone App for Dietary Intake Assessment: Comparison with a Food Frequency Questionnaire

**DOI:** 10.2196/resprot.5782

**Published:** 2016-11-02

**Authors:** Luis Maria Bejar, Brett Northrop Sharp, María Dolores García-Perea

**Affiliations:** ^1^ Department of Preventive Medicine and Public Health University of Seville Seville Spain; ^2^ Virgen Rocio University Hospital Seville Spain; ^3^ Virgen Macarena University Hospital Seville Spain

**Keywords:** dietary assessment, mobile phone application, food frequency questionnaire, epidemiological methods

## Abstract

**Background:**

There is a great necessity for new methods of evaluation of dietary intake that overcome the limitations of traditional self-reporting methods.

**Objective:**

The objective of this study was to develop a new method, based on an app for mobile phones called e-EPIDEMIOLOGY, which was designed to collect individual consumption data for a series of foods/drinks, and to compare this app with a previously validated paper food frequency questionnaire (FFQ).

**Methods:**

University students >18 years of age recorded the consumption of certain foods/drinks using e-EPIDEMIOLOGY during 28 consecutive days and then filled out a paper FFQ at the end of the study period. To evaluate the agreement between the categories of habitual consumption for each of the foods/drinks included in the study, cross-classification analysis and a weighted kappa statistic were used.

**Results:**

A total of 119 participants completed the study (71% female, 85/119; 29% male, 34/119). Cross-classification analysis showed that 79.8% of the participants were correctly classified into the same category and just 1.1% were misclassified into opposite categories. The average weighted kappa statistic was good (κ=.64).

**Conclusions:**

The results indicate that e-EPIDEMIOLOGY generated ranks of dietary intakes that were highly comparable with the previously validated paper FFQ. However, it was noted that further testing of e-EPIDEMIOLOGY is required to establish its wider utility.

## Introduction

Traditional self-reporting methods that evaluate dietary intake, such as dietary registries and 24-hour recall questionnaires (short-term methods), and food frequency questionnaires (FFQs; long-term instruments) present important limitations [1-4]. Short-term tools allow for the collection of data that include quantities of all foods/drinks consumed by a person during a certain number of days. Dietary registries that require weighing of foods are time-consuming and create a great deal of work for study participants, which can lead to deviations from normal food intake (especially underestimation of quantities), as well as low rates of participation and compliance. The use of 24-hour recall questionnaires requires trained personnel and are short-term memory dependent. In order to determine habitual dietetic intake (the long-term mean consumption of foods/drinks) using these short-term tools, it would also be necessary to repeat these measures multiple times, which would only worsen the problems inherent to these procedures. Long-term recall methods such as FFQs allow information to be collected regarding the consumption of a series of foods/drinks over prolonged periods of time (weeks or months), classifying a person according to the consumption category applied to each of the foods/drinks considered. FFQs depend mostly on the memory of the subject being interviewed, and these questionnaires do not take into account intrapersonal variation in the recording of daily food consumption during the time period of the study, nor do they allow precise estimation of food portion size. Despite these limitations, FFQs are the most practical, accessible, and commonly utilized tools in research to determine habitual dietary intake [2,5,6]. One inherent limitation to most FFQs is that they are paper-based. As a result, errors such as skipped questions or multiple marks are common, and incorporating complex skip patterns, a broad and varying number of portions size options, and extensive food and portion-size graphics is challenging [7]. Both long-term and short-term tools employ traditional techniques (paper and pen) to collect information, with posterior manual introduction for statistical analyses, which increases research costs and time consumption considerably [3,8]. For these reasons, improvement upon traditional methods for the determination of dietary intake remains one of the most important challenges in nutritional epidemiology [5,8-10]. Improvement of self-reporting that contributes to greater precision in the measurement of habitual dietary intake would represent a considerable boon for researchers, as well as for society as a whole, considering the important repercussions that the results and conclusions of these studies can have on the general population.

Traditional self-reporting techniques that evaluate dietary intake need to be replaced by new solutions, or nutritional research and treatments for nutritional problems will remain restricted and deficient [11]. Web-based FFQs offer straightforward solutions to the limitations of paper FFQs, and several examples of computer-administered FFQs exist in the published literature [12-16]. Additionally, certain dietary registries and 24-hour recall mobile phone apps have been developed recently that could reduce the limitations of these methods, with promising results [8-10,17,18].

The use of the Internet on mobile phones is widespread in Spain, with 83% of all Spaniards having accessed the Internet using their mobile phones within the last three months. This usage is even more extended in Spaniards between the ages of 16 and 24, with 92.6% accessing the Internet via their mobile phones in this same time period [19]. This broad usage facilitates the introduction of new methods of evaluation of dietary intake that include mobile technology. These new technologies need to be developed according to different local conditions, and evaluated with objective measures [10].

The objective of this study was to develop a new method, based on an application for mobile phones called e-EPIDEMIOLOGY, that was designed to collect individual consumption data about a series of foods/drinks, and to compare data recorded using this tool with that obtained through a previously validated paper FFQ.

## Methods

### Study Sample

This study was performed among medical and pharmaceutical students at the University of Seville (Andalusia, Spain, Southern Europe). Different events were organized at both faculties, during which the research team personally presented the project to the students. At the end of each presentation, interested students and those that fit inclusion criteria signed up for a personal interview. Of the 183 students that were interested, 136 were eligible and were signed up for the interview, in which the study protocol was explained. A total of 120 students decided to participate in the study. Of these, 119 completed both the e-EPIDEMIOLOGY app and the paper FFQ. The period of participant recruitment spanned from October, 2014 to June, 2016. The inclusion criteria were the following: (1) being a University of Seville student from the Medical or Pharmaceutical Schools; (2) being over 18 years of age; and (3) owning a mobile phone with access to the Internet and an Android operating system. As an incentive, all participants were entered into a raffle for a tablet at the conclusion of the study. The study was performed according to directives established in the Helsinki Declaration and the Biomedical Research Law [20], and all procedures on human beings were approved by the Research Ethics Committee at the University of Seville. Written informed consent was obtained from all participants.

### The e-EPIDEMIOLOGY Mobile Phone App

Participants downloaded the e-EPIDEMIOLOGY app to their personal mobile phones. This app permitted the recording of each participant’s daily consumption of a series of the foods/drinks selected for the study. At the end of each day, a notice would appear on the participant’s mobile phone, informing them that it was time to use the app. At that time, the participant could access the app and register the number of standard portions that had been consumed during that day, for each of the foods/drinks included in the study. The list of foods/drinks appeared every day in the same order to facilitate completion of the app. This list consisted of 12 items which referred to 10 different foods/drinks: fruit, vegetables, legumes, chicken/turkey, fish, red meat (lamb, beef, and pork), soft drinks, sweets, prepared foods, and alcoholic beverages (Multimedia Appendix 1).

These items were selected for the study because they provide a wide range of consumption patterns, from *daily* to *sporadic*, for the population [5]. These foods were also considered to be markers for healthy (fruits, vegetables, legumes, and fish) and unhealthy (soft drinks, sweets, and prepared foods) dietary habits [21]. When accessing the first food/drink on the list, the number of standard portions of this food/drink consumed throughout the day was introduced. The *Next* button was then pressed to go on to the following item, in order to record all foods/drinks consumed that day (Multimedia Appendix 2). After finishing the task on e-EPIDEMIOLOGY, the data was automatically saved and sent to the research administrator’s website via Wi-Fi or 3G/4G, after which time the user could not access or change answers on the questionnaire.

The app used to register daily consumption of selected foods/drinks was based on a questionnaire elaborated upon using the FFQ from the European Health Survey (Multimedia Appendix 1) [22]. Standardized portions were added after testing a previous prototype of e-EPIDEMIOLOGY (results not published) and were obtained from an FFQ validated for the Spanish population [23]. The app also allows for registry of other lifestyle habits (hours of sleep, oral hygiene, physical activity, and tobacco consumption). The app recorded this information using a different questionnaire with 11 items, which were also based on validated instruments from the European Health Survey [22].

### Anthropomorphic Measurements

Researchers used the personal interview to both explain the study protocol and collect anthropomorphic data using a standard procedure. Height was measured in centimeters (cm), with a precision of 0.5 cm, and weight was measured in kilograms (kg), with a precision of 0.1 kg (wearing lightweight clothing, with shoes off and pockets empty). Using these data, body mass index (BMI; kg/m^2^) was calculated using categories defined by the World Health Organization [24].

### Procedure

All participants completed a questionnaire during the personal interview, in which demographic data was collected (date of birth, gender, birthplace, current place of residence, and employment). Participants were instructed in the use of e-EPIDEMIOLOGY with a personal demonstration of how to use the app, as well as an estimation of standardized portion sizes, and were reminded to maintain their habitual diet. The recording of food/drink intake was to be completed during 28 consecutive days using the app. Participants were recruited to the study during the entire period of research, so that all seasons, as well as days of the month and week, were included in the sample. As a reference, a paper FFQ was filled out at the end of the study period, through personal interviews and at the convenience of the participants. During the personal interviews, the participants were also asked how much time, on average, was necessary to complete the task each day. Participants could choose from one of the following options: *approximately 1 minute per day*, *approximately 2 minutes per day*, *approximately 3 minutes per day*, *approximately 4 minutes per day*, or *approximately 5 minutes or more per day*. Almost all (94.1%; 112/119) of the participants selected the option *approximately 1 minute per day* and the remaining 5.9% (7/119) chose *approximately 2 minutes per day*. Thus, the time necessary to complete e-EPIDEMIOLOGY was approximately one minute per day. Both methods asked about food/drink intake over a period of 28 days, and in order to make comparisons about the usefulness of each tool, it was desirable to keep food/drink records during the same period of time with each method [25]. The paper FFQ was based on a previously validated questionnaire used in the European Health Survey (Multimedia Appendix 3) [22]. Standardized portion sizes were obtained from an FFQ validated for the Spanish population [23]. Both of the questionnaires used in the app and the paper FFQ had the same items (Multimedia Appendices 1 and 3); the only difference being that the e-EPIDEMIOLOGY questionnaire refers to daily consumption while the paper FFQ refers to consumption during the previous 28 days.

All of the personal data collected in this study remained anonymous and confidential, and were treated according to current Spanish legislation [26]. To that end, each participant was assigned a personal alphanumeric code, so that no one (including the researchers) could link personal information to the results obtained. The code was introduced the first time the participant accessed the app, and when completing the demographic questionnaire and paper FFQ, for organizational purposes.

### Codification and Revision of Data

For each participant, the data collected from the paper FFQ for each of the 10 foods/drinks were categorized. The frequency of consumption of food/drink items was categorized into six subgroups, ranging from *Less than once a week* to *3 times or more a day* (Multimedia Appendix 3). For the same foods/drinks, the data from the 28 days using e-EPIDEMIOLOGY were recorded as daily consumption. These data were transformed in order to include them in one of the same categories of habitual consumption included in the FFQ. This analysis was made possible because both the paper FFQ and e-EPIDEMIOLOGY used the same standardized portion sizes. For example, a participant consumed an average of 0.25 standard rations of fish daily during 28 days using e-EPIDEMIOLOGY; this average consumption represents 1.75 standard portions per week (0.25x7=1.75), which would be classified in the category *Once or twice a week*.

The data collected from the paper FFQ were manually introduced into the database by the research team. These results were then reviewed in order to avoid data entry errors. Data collected from e-EPIDEMIOLOGY were saved without modifications in a separate database. Subsequently, one set of data was removed due to an obvious inconsistency: one participant had registered the consumption of 200 standardized portions of legumes in one day.

### Statistical Analyses

Due to the lack of agreement on the best way to present results from comparison studies, it is necessary to use more than one statistical method, in order to give credence to the results. In this study, cross-classification analysis and the weighted kappa statistic were used. To assess agreement, subjects were classified into categories of intake by e-EPIDEMIOLOGY and the reference method, and the percentage of subjects correctly classified into the same category, and misclassified into different categories, was calculated. Using cross-classification, the percentages misclassified clearly illustrate the likely impact of measurement error; however, the percentage of agreement will include agreement that can be accounted for by chance. The weighted kappa statistic is a summary measure of cross-classification that takes into account the agreement expected by chance, and has the added advantage over the kappa statistic in that it also takes into account the degree of misclassification. However, both the cross-classification analysis and the weighted kappa statistic are still dependent on the number of categories used [27]. In order to limit this dependence, the six original categories were reorganized into three (Category 1: *Less than once a week* and *Once or twice a week*; Category 2: *3-4 times a week* and *5-6 times a week*; Category 3: Once or twice a day and *3 times or more a day*), in order to apply criteria defined by Masson et al [27] to evaluate agreement and misclassification. The interrater agreement of two assessment methods was analyzed by weighted kappa statistic [28], assigning partial credit to scores using the Stata prerecorded weights. If there was complete agreement, a weight of 1.00 was assigned. Slight disagreements (off by one) were given a weight of .50, and a weight of .00 was assigned if there was a complete disagreement. Values of kappa >.80 indicate very good agreement; between .61 and .80 indicate good agreement; .41 to .60 indicate moderate agreement; .21 to .40 indicate fair agreement; and <.20 indicate poor agreement [27]. All statistical analyses were performed using STATA version MP 13.1 (Stata Corp LP, Texas, USA) and a  *P* value <.05 was considered statistically significant [29].

## Results

A total of 120 individuals participated in the study, but one participant did not complete the app and the FFQ. This individual’s data were not used for posterior analyses. Of the 119 participants who completed the study, 93 individuals completed the app every day, 15 completed the app 26 days, 1 completed the app 25 days, 9 completed the app 24 days, and 1 completed the app 20 days (Table 1).

Among the participants, the mean age was 21.9 years (standard deviation [SD] 3.2). The sample was 71.4% (85/119) female and 28.6% (34/119) male, and a minority (15.1%, 18/119) of participants were smokers. Less than one third (29.4%, 35/119) of respondents performed 150 minutes or more of moderate-intensity physical activities per week. The mean BMI was 22.3 kg/m^2^ (SD 3.1), with 72.3% of participants in the healthy weight range (86/119; BMI 18.5-24.9), 16.8% being overweight (20/119; BMI 25.0-29.9), 2.5% obese (3/119; BMI >30.0), and 8.4% underweight (10/119; BMI <18.5) (Table 1).

The mean percentage of individuals correctly classified into the same category was 79.8% (ranging from 73.9% for vegetables to 84.9% for prepared foods), the mean percentage of individuals misclassified into an adjacent category was 19.1% (ranging from 15.1% for prepared foods to 26.1% for vegetables), and the mean percentage of individuals misclassified into an opposite category was 1.1% (ranging from 0% for vegetables, fish, and prepared foods to 3.4% for sweets; Table 2).

**Table 1:** Characteristics of participants in the study.

Characteristics		n (%)	mean (SD)
Participants who completed the study		119	
**Number of days completed through the app**			
	28 days	93 (78.2)	
	26 days	15 (12.6)	
	25 days	1 (0.8)	
	24 days	9 (7.6)	
	20 days	1 (0.8)	
Age in years			21.9 (3.2)
**Gender**			
	Female	85 (71.4)	
	Male	34 (28.6)	
**Smoking status**			
	No	101 (84.9)	
	Yes	18 (15.1)	
**Physical activity status**			
	150 minutes or more/week	35 (29.4)	
	Less than 150 minutes/week	84 (70.6)	
**BMI in kg/m^2^**			22.3 (3.1)
	Underweight	10 (8.4)	
	Normal range	86 (72.3)	
	Overweight	20 (16.8)	
	Obesity	3 (2.5)	

**Table 2:** Cross-classification analysis derived from e-EPIDEMIOLOGY and the paper FFQ.

Comparison	Agreement (%)		
	Same category	Adjacent category	Extreme category
Fruit	79.8	18.5	1.7
Vegetables	73.9	26.1	0.0
Legumes	83.2	16.0	0.8
Chicken/turkey	80.7	16.8	2.5
Fish	76.5	23.5	0.0
Red meat	79.8	19.3	0.8
Soft drinks	79.0	20.2	0.8
Sweets	79.8	16.8	3.4
Prepared foods	84.9	15.1	0.0
Alcoholic beverages	80.7	18.5	0.8
Average	79.8	19.1	1.1

The average weighted kappa statistic was good (κ=.64). The weighted kappa statistic values showed good agreement for fruit, vegetables, chicken/turkey, red meat, soft drinks, sweets, and alcoholic beverages (κ=.61 to .70) and moderate agreement for legumes, fish, and prepared foods (κ=.52 to .55; Table 3).

**Table 3:** Percentage agreement, percentage expected agreement, and weighted kappa statistic derived from e-EPIDEMIOLOGY and the paper FFQ.

Comparison	Agreement (%)	Expected agreement (%)	Weighted kappa	*P* value
Fruit	89.1	63.2	0.70	<.001
Vegetables	87.0	59.8	0.68	<.001
Legumes	91.2	81.7	0.52	<.001
Chicken/turkey	89.1	66.3	0.68	<.001
Fish	88.2	73.9	0.55	<.001
Red meat	89.5	72.7	0.61	<.001
Soft drinks	89.1	64.8	0.69	<.001
Sweets	88.2	60.9	0.70	<.001
Prepared foods	92.4	83.8	0.53	<.001
Alcoholic beverages	89.9	67.1	0.69	<.001
Average	-	-	0.64	-

## Discussion

### Principal Findings

The present study puts forth the development of a new method for the determination of habitual dietary intake using mobile technologies, and its comparison with a previously validated paper FFQ. Recently, certain short-term methods that use mobile technologies have been developed [8-10,17,18]. However, until now, no long-term instruments had been developed for evaluating habitual dietary intake, benefitting from mobile technologies and serving as an alternative to traditional FFQs. This new method, based on an app for mobile phones called e-EPIDEMIOLOGY, is not intended to determine the total food consumption of an individual nor the exact quantity consumed of a selected food/beverage. There are different tools, such as dietary registries or 24-hour recalls, which serve that purpose [1-4]. This method using e-EPIDEMIOLOGY was designed to record the amount of selected foods/drinks consumed throughout each day during the study period; data which can later be used to calculate the average consumption of said items in that period. This process then allows for classification of participants into distinct categories of habitual consumption of selected foods/drinks. The app can also be used to identify potential deficits in nutrient consumption, to analyze possible associations with risks for chronic diseases, and to evaluate the effectiveness of personalized measures that promote healthy lifestyle changes [7]. Although this method allows for the classification of individuals into categories (much like an FFQ), it is basically a simplified 24-hour food recall, repeated many times (once per day) during the study period of 28 days. Ultimately both methods (e-EPIDEMIOLOGY and FFQ) are very different and therefore present different measurement errors, due, for example, to the fact that dependence on the memory of participants in both methods is different (e-EPIDEMIOLOGY data collection is performed daily, while the collection of data with paper FFQs refers to the last 28 days), or that e-EPIDEMIOLOGY allows for daily intrapersonal variability in the collection of data regarding the consumption of foods/drinks (which is not possible with a paper FFQ).

Cross-classification analysis showed that 79.8% of the participants were correctly classified into the same category and just 1.1% were misclassified into an opposite category. The average weighted kappa statistic was good (κ=.64), with values >.55 for 8 of the 10 foods/drinks selected for the study. These results indicate that e-EPIDEMIOLOGY generates ranks of dietary intakes that are highly comparable with the previously validated paper FFQ [13], according to Masson’s criteria [27]. However, it was noted that further testing of e-EPIDEMIOLOGY is required to establish its wider utility [13,30]. While e-EPIDEMIOLOGY demonstrated good agreement with the paper FFQ, some disagreement was observed between the two instruments (cross-classification analysis showed that 19.1% of the participants were incorrectly classified into an adjacent category and 1.1% were misclassified into an opposite category). Multiple factors could have contributed to the discrepancies observed between the two methods. For each of the foods/drinks considered, both methods used the same question to measure the frequency of consumption. For example, both ask, *“How many portions of fish have you eaten? (1 portion = approx. 150 g)”*. Consequently, both methods present the same difficulties in the precise estimation of portion size, given that standardized serving sizes are used in both.

The difference between the methods lies in the timing of responses: e-EPIDEMIOLOGY requires that questions are answered at the end of each day during the study period, while the paper FFQ is completed at the end of 28 days. For this reason, e-EPIDEMIOLOGY permits daily collection of information, while an FFQ only allows for the collection of information at the end of the study period. This shortened time frame minimizes the dependence on the memory of the participant using e-EPIDEMIOLOGY in comparison to the FFQ, considering the fact that the recollection of past consumption of foods can be influenced by more recent food consumption [3]. Additionally, e-EPIDEMIOLOGY allows for daily intrapersonal variability in the collection of data on the consumption of foods/drinks. Among university students, who comprised the study sample, dietary intake is variable from day to day, with sporadic changes in food intake (skipping meals, snacking, school events that interfere with meal times), as well as frequent dining out. These aspects interfere with the precise determination of habitual dietary intake [17], especially in the case of FFQs, when data is collected only once at the end of an extended time period. Repeated applications of traditional short-term instruments, such as dietary registries and 24-hour recalls, can modify habitual intake due to the excessive workload for participants. Any tool that provides a simple method that facilitates the collection of data regarding dietary intake, without changing behavior, is an important advancement in nutritional epidemiology [17]. Despite repeated use, the modification of habitual intake seems unlikely via the use of e-EPIDEMIOLOGY, due to the reduced workload that using this app presents (one minute per day).

Interviewer-administration of 24-hour recall questionnaires or FFQs, versus self-administration, may decrease the accuracy of dietary intake reporting [31,32]. Psychological factors, among others, may have contributed to this underreporting, such as social desirability and a fear of negative evaluation [32]. If the data collection method was administered by an interviewer, participants with a high drive for social desirability were provided with an opportunity to please the interviewer. Conversely, the interviewer may have provoked underreporting of the consumption of food in those with a fear of negative evaluation [32]. Several studies suggest that underreporters are more likely to estimate low intake of foods perceived as unhealthy or undesirable (eg, sweets, fats, and snacks) than those perceived as healthy (eg, fruits, vegetables, and reduced fat products) [30,33,34]. Some of the characteristics of e-EPIDEMIOLOGY, such as asynchrony [35-38] and the ease with which privacy can be maintained [39], have made it possible to collect data anonymously on the Internet. This factor could contribute to reducing the problem of underestimation, mainly with those foods/drinks that are socially considered unhealthy, as this would minimize the effect of the psychological factors previously mentioned.

In their most simple applications, paper FFQs match Web-based FFQs; this allows for the flexibility of using either a paper or computerized questionnaire interchangeably, but the benefits of computer administration are limited to direct data entry, real-time error checking, and rapid analysis [40]. Other advantages include reducing paper waste and postage costs, and optimizing the space, security, and organization required for paper file storage [12]. In this study, it was considered that the potential disadvantages of developing a Web-based FFQ (in comparison with a paper-based FFQ) outweighed its potential benefits, keeping in mind two inherent characteristics of this study: the paper FFQ used was very short and simple (containing only 12 items), and the sample was comprised of students from the Medical and Pharmacy Schools of the University of Seville. The simplicity of the paper FFQ reduced the chance for errors, the amount of paper consumed, and storage space issues. Relatively easy access to the sample population made it possible to complete the paper FFQ in person, making it unnecessary to send it via mail. In this case, the costs associated with data entry were minimal compared with the potential costs of developing a Web-based FFQ.

For research, clinical practice, and policy determination, a great need exists for accurately determining dietary intake. However, current methods of self-reporting present limitations that are amply described in the scientific literature [1-4]. Due to these limitations, results obtained from these inaccurate scientific methods can lead to inaccurate conclusions and decision-making. Emerging alternatives for the determination of dietary intake include digital photography, chewing and swallowing monitors, and wrist motion detectors that count plate-to-mouth motion [41-44]. Some authors argue that more research is needed to develop these and other more objective and accurate tools. In addition, long-term funding should be made available for the measurement of dietary intake, whereby consumption can be measured over long periods of time. Meanwhile, the use of decidedly inaccurate instruments to measure dietary intake needs to be discontinued [45]. A necessity exists for the development of better methods that can eventually replace current long-term self-reporting methods, and more resources should be directed to that end. Until these long-term alternatives are available, new technologies for self-reporting methods can, and should, be developed and utilized. Thus, further research to improve both short-term and long-term self-reporting methods (not only for clinical applications, but also for investigations) is well motivated [7]. These new tools, developed using new technologies such as e-EPIDEMIOLOGY, should be validated with objective studies that allow for the confirmation of an improvement over the traditional methods upon which they are based.

### Limitations

The main limitation of this study is the fact that e-EPIDEMIOLOGY was compared to an FFQ (not validated). Another limitation of this study is the possible rate of nonresponse. Some of the characteristics of these types of mobile technologies, such as asynchrony [35-38], the ease with which privacy can be maintained [39], and the light workload for the participants (1 minute per day), helped to increase participation and could have contributed to the minimization of nonresponse rates. Young people have expressed their preference for methods of dietary intake evaluation that utilize new technologies, as they can easily be incorporated into their lifestyles, and are more amenable than traditional paper-based methods [9,17]. The possible limitation presented by the rate of nonresponse was minimized, as no statistically significant differences were found in any of the variables studied (age, gender, tobacco consumption, physical activity, and BMI), after analyzing the basic characteristics of responders and nonresponders.

Another possible limitation of the current study is that the participants involved were students. The majority of participants were also women (which is a reflection of the proportion of male and female students enrolled in the Schools of Medicine and Pharmacy at the University of Seville) and were, therefore, representative of a convenient sample rather than a nationally representative sample.

Another possible limitation lies in the fact that access to these technologies is not universal, excluding especially vulnerable groups, such as students from poorer social strata. In the environment in which this study was performed, the percentage of students with mobile phones with Internet access was very high, which minimized this possible limitation [19].

### Future Studies

A validation study has been planned in which both methods (e-EPIDEMIOLOGY and paper FFQs), will be compared to a 3-7 day weighed food record. This approach will help to more thoroughly evaluate the potential validity of e-EPIDEMIOLOGY as a research tool for the determination of habitual dietary intake. Evaluation of e-EPIDEMIOLOGY is also planned in different sociodemographic groups, and will entail modifying the follow-up time, reducing from daily data input to input 2-3 times per week, and varying the foods/drinks selected. Another line of study would be to analyze the impact of factors that can affect the validity of data collected with e-EPIDEMIOLOGY, such as age, gender, employment, and health-related behavior (tobacco consumption, physical activity, and BMI). In future validation studies of e-EPIDEMIOLOGY, a third version of the app will be used (the second version is currently in use) which includes several improvements, such as an adaptation to iOS (which will help increase the sample size), and the inclusion of photographs to help participants estimate portion size.

### Conclusions

In conclusion, this study of young adults generated good agreement with a previously validated paper-based FFQ. A variety of analyses, combined with the ease of use of e-EPIDEMIOLOGY, indicated the utility of the method based on this app for classifying individuals according to their consumption of the foods/drinks selected for the study, and is potentially valuable for use in other epidemiological studies as an alternative to paper FFQs [13]. Due to the growing popularity of mobile phones among young adults, the e-EPIDEMIOLOGY app is likely to be accepted by this population, and could reduce some of the inherent limitations present in paper FFQs, such as dependence on the memory of participants and the impossibility of reflecting intrapersonal variability in daily consumption of foods/drinks. However, it was noted that further testing of e-EPIDEMIOLOGY is required to establish its wider utility [13,30].

## Appendix

Multimedia Appendix 1 Questionnaire used in e-EPIDEMIOLOGY, with weights/measurements of standardized portions of selected foods/drinks.

Multimedia Appendix 2 Twelve screen captures of the e-EPIDEMIOLOGY app.

Multimedia Appendix 3 Questionnaire utilized for paper FFQ, with weights/measurements of standardized portions of selected foods/drinks.

## Abbreviations

BMI: body mass index
cm: centimeter
FFQ: food frequency questionnaire
kg: kilogram
SD: standard deviation
