# Supplementary material for: The e-EPIDEMIOLOGY Mobile Phone App for Dietary Intake Assessment: Comparison with a Food Frequency Questionnaire
Source: JMIR Res Protoc. 2016 Nov 2;5(4):e208. doi: 10.2196/resprot.5782 (PMC5112366; doi:10.2196/resprot.5782)
Supplement: Multimedia Appendix 1 [file resprot_v5i4e208_app1.pdf]

**Multimedia Appendix 1. Questionnaire used in e-EPIDEMIOLOGY, with weights / measurements of standardized portions of selected foods/drinks.**

| |
|---|
| 1. How many pieces of fruit have you eaten today? (1 piece = approx. 100 g) (Include fresh-squeezed juice (1 portion = approx. 200 ml)) |
| 2. How many portions of vegetables have you eaten today? (1 portion = approx. 150 g) |
| 3. How many portions of legumes (lentils, garbanzos, beans, etc.) have you eaten today? (1 portion = approx. 60 g) |
| 4. How many portions of chicken/turkey have you eaten today? (1 portion = approx. 150 g) |
| 5. How many portions of fish have you eaten today? (1 portion = approx. 150 g) |
| 6. How many portions of red meat (beef, pork, lamb) have you eaten today? (1 portion = approx. 150 g) |
| 7. How many servings of soft drinks have you had today? (1 serving = approx. 250 ml) |
| 8. How many portions of commercially produced sweets (not home-made) (cookies/pastries) have you eaten today? (1 piece = approx. 100 g) |
| 9. How many portions of prepared/frozen foods have you eaten today (croquettes, pizza, etc.)? (1 portion = approx. 80 g) |
| 10. Have you consumed alcoholic beverages today? |
| 11. What kind of alcoholic beverage have you consumed? |
| 12. How many servings of beer/wine/spirits or mixed drinks have you consumed today? (1 serving of beer = approx. 200 ml / 1 glass of wine = approx. 100 ml / 1 serving of spirits or mixed drinks = approx. 50 ml (of alcohol)) |
