# Supplementary material for: The e-EPIDEMIOLOGY Mobile Phone App for Dietary Intake Assessment: Comparison with a Food Frequency Questionnaire
Source: JMIR Res Protoc. 2016 Nov 2;5(4):e208. doi: 10.2196/resprot.5782 (PMC5112366; doi:10.2196/resprot.5782)
Supplement: Multimedia Appendix 2 [file resprot_v5i4e208_app2.pdf]

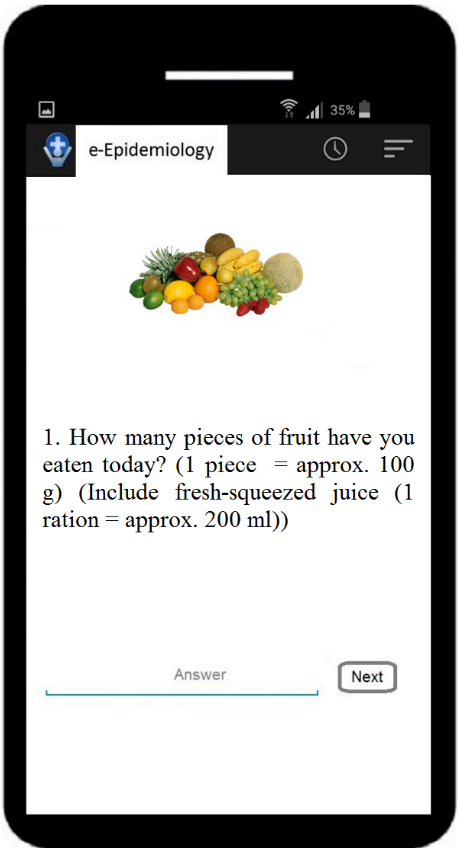
35%
e-Epidemiology
1. How many pieces of fruit have you eaten today? (1 piece = approx. 100 g) (Include fresh-squeezed juice (1 ration = approx. 200 ml))
Answer
Next

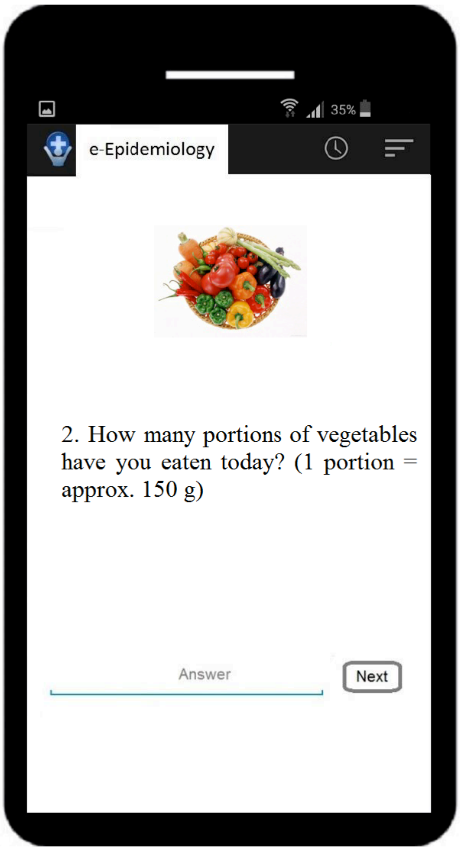
35%
e-Epidemiology
2. How many portions of vegetables have you eaten today? (1 portion = approx. 150 g)
Answer
Next

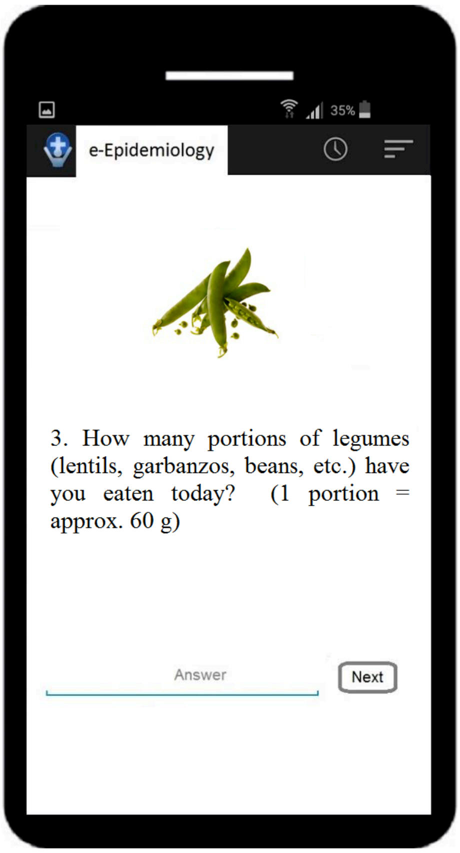
35%
e-Epidemiology
3. How many portions of legumes (lentils, garbanzos, beans, etc.) have you eaten today? (1 portion = approx. 60 g)
Answer
Next

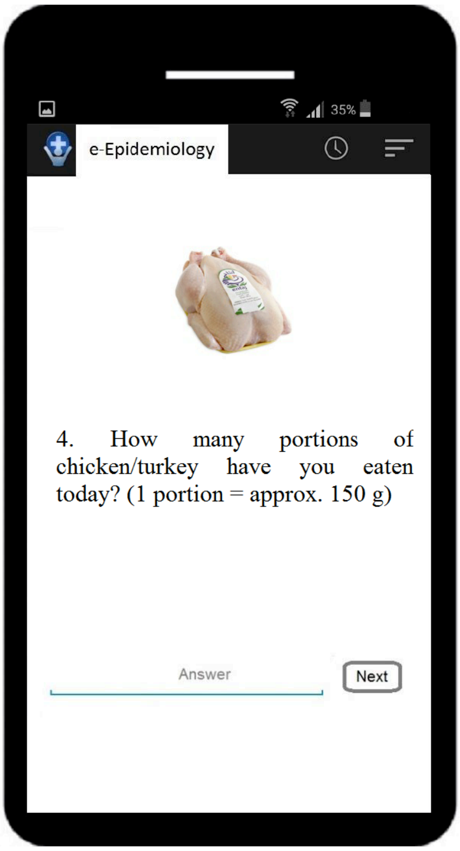
35%
e-Epidemiology
4. How many portions of chicken/turkey have you eaten today? (1 portion = approx. 150 g)
Answer
Next

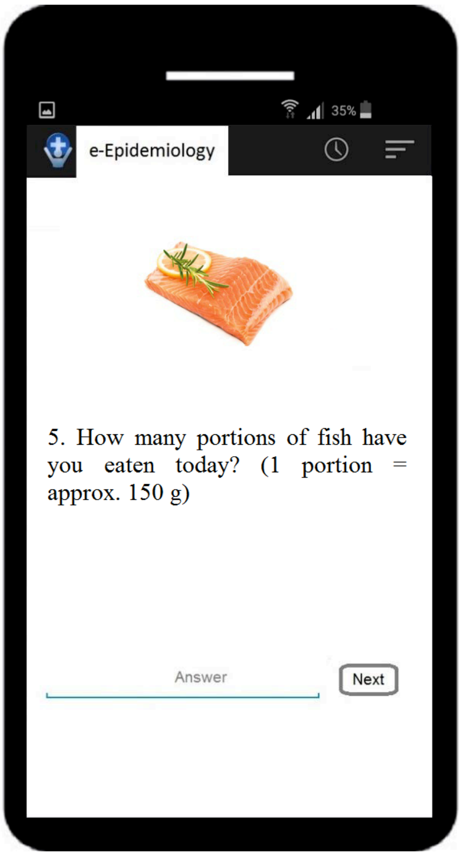
35%
e-Epidemiology
5. How many portions of fish have you eaten today? (1 portion = approx. 150 g)
Answer
Next

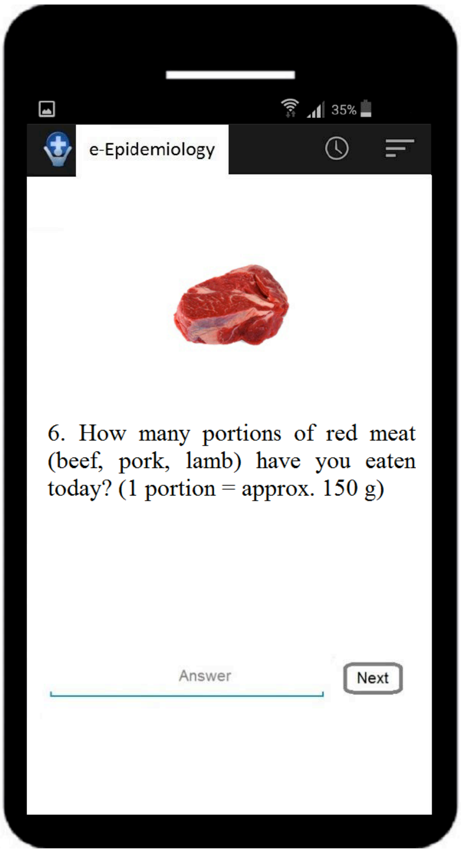
35%
e-Epidemiology
6. How many portions of red meat (beef, pork, lamb) have you eaten today? (1 portion = approx. 150 g)
Answer
Next

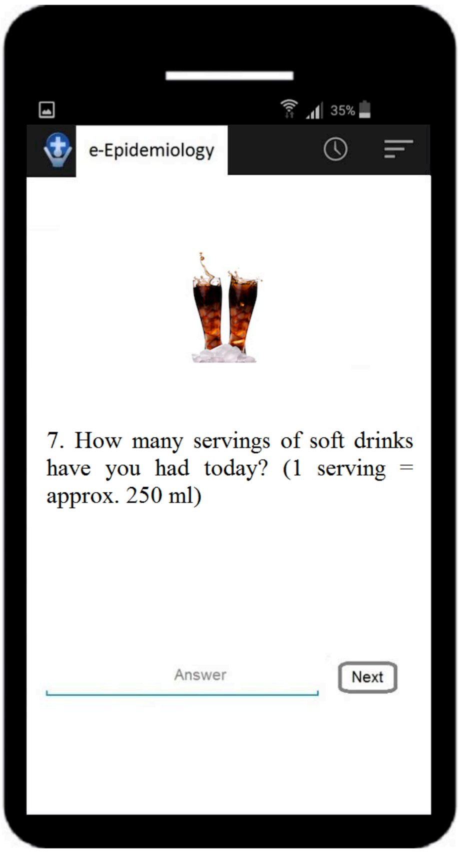
35%
e-Epidemiology
7. How many servings of soft drinks have you had today? (1 serving = approx. 250 ml)
Answer
Next

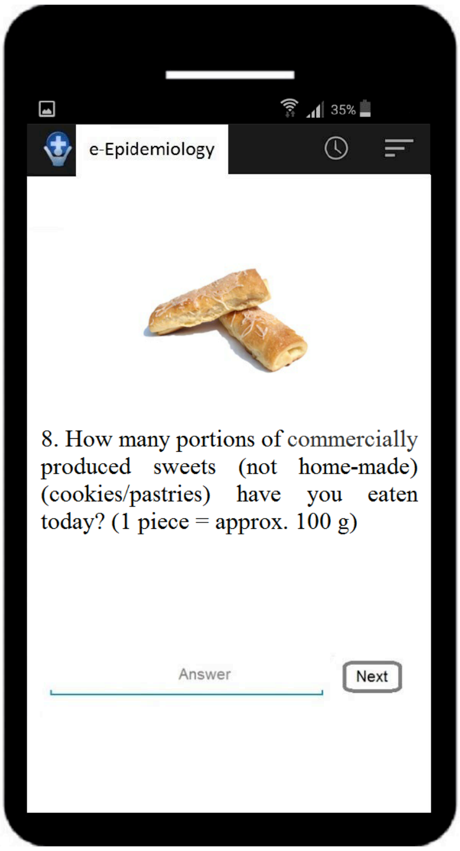
35%
e-Epidemiology
8. How many portions of commercially produced sweets (not home-made) (cookies/pastries) have you eaten today? (1 piece = approx. 100 g)
Answer
Next

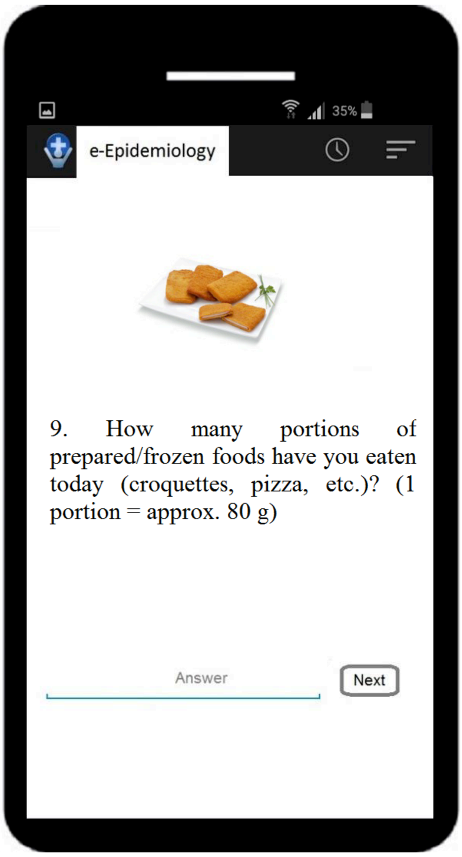
35%
e-Epidemiology
9. How many portions of prepared/frozen foods have you eaten today (croquettes, pizza, etc.)? (1 portion = approx. 80 g)
Answer
Next

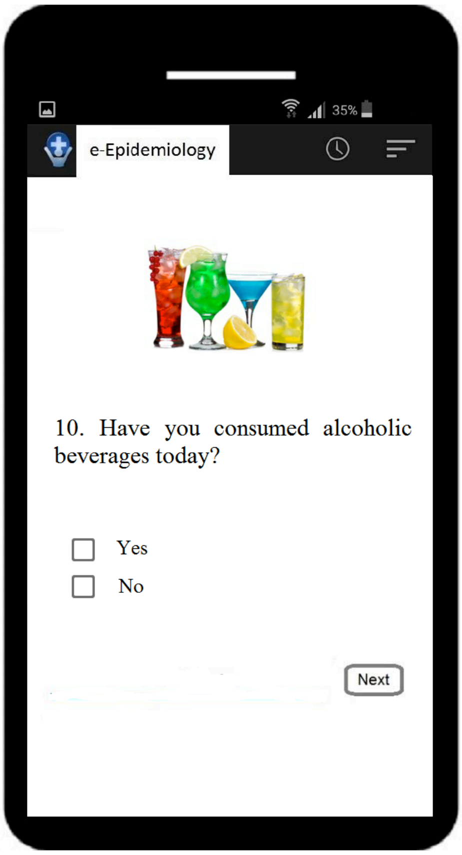
35%
e-Epidemiology
10. Have you consumed alcoholic beverages today?
Yes
No
Next

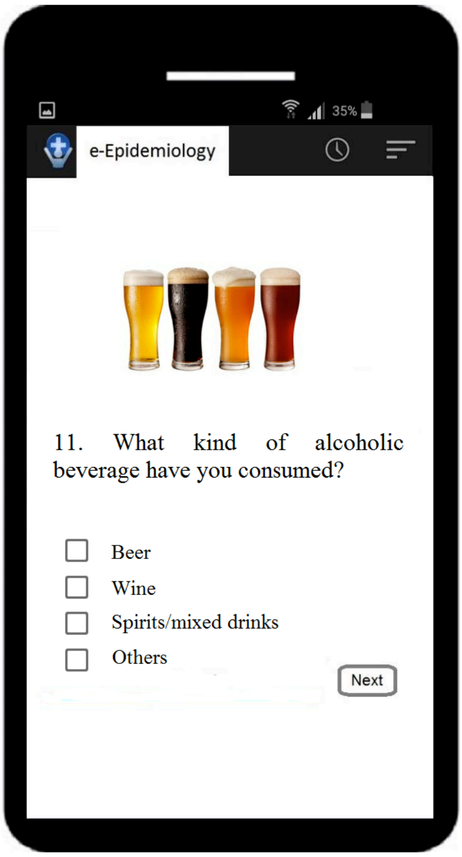
35%
e-Epidemiology
11. What kind of alcoholic beverage have you consumed?
Beer
Wine
Spirits/mixed drinks
Others
Next

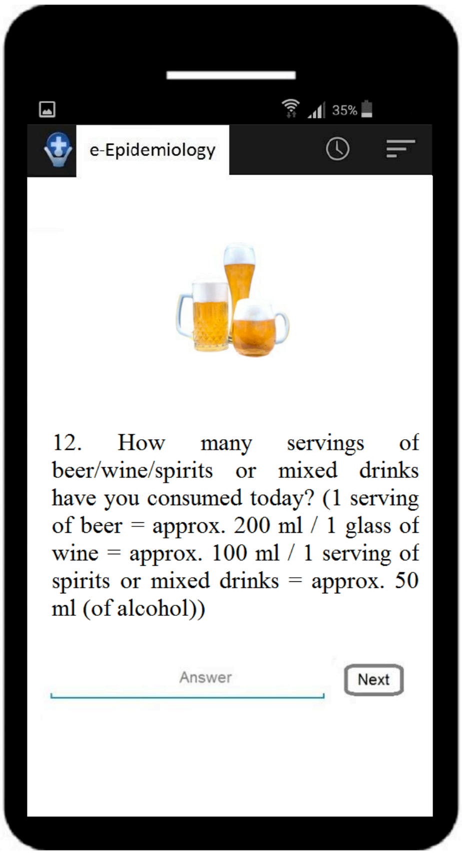
35%
e-Epidemiology
12. How many servings of beer/wine/spirits or mixed drinks have you consumed today? (1 serving of beer = approx. 200 ml / 1 glass of wine = approx. 100 ml / 1 serving of spirits or mixed drinks = approx. 50 ml (of alcohol))
Answer
Next
