# Supplementary material for: The e-EPIDEMIOLOGY Mobile Phone App for Dietary Intake Assessment: Comparison with a Food Frequency Questionnaire
Source: JMIR Res Protoc. 2016 Nov 2;5(4):e208. doi: 10.2196/resprot.5782 (PMC5112366; doi:10.2196/resprot.5782)
Supplement: Multimedia Appendix 3 [file resprot_v5i4e208_app3.pdf]

Multimedia Appendix 3. Questionnaire utilized for paper FFQ, with weights/measurements of standardized portions of selected foods/drinks.

1. How many pieces of fruit did you habitually consume in the last 28 days?<br>(1 piece = approx. 100 g) (Include fresh-squeezed juice (1 portion = approx. 200 ml))	Categories[a]
2. How many portions of vegetables did you habitually consume in the last 28 days? (1 portion = approx. 150 g)	Categories[a]
3. How many portions of legumes (lentils, garbanzos, beans, etc.) did you habitually consume in the last 28 days? (1 portion = approx. 60 g)	Categories[a]
4. How many portions of chicken/turkey did you habitually consume in the last 28 days? (1 portion = approx. 150 g)	Categories[a]
5. How many portions of fish did you habitually consume in the last 28 days? (1 portion = approx. 150 g)	Categories[a]
6. How many portions of red meat (beef, pork, lamb) did you habitually consume in the last 28 days? (1 portion = approx. 150 g)	Categories[a]
7. How many servings of soft drinks did you habitually consume in the last 28 days? (1 serving = approx. 250 ml)	Categories[a]
8. How many portions of commercially produced sweets (not home-made) (cookies/pastries) did you habitually consume in the last 28 days? (1 piece = approx. 100 g)	Categories[a]
9. How many portions of prepared/frozen foods have you habitually eaten (croquettes, pizza, etc.) in the last 28 days? (1 portion = approx. 80 g)	Categories[a]

10. Have you consumed alcoholic beverages in the last 28 days?	☐ Yes<br>☐ No
11. What kind of alcoholic beverages have you consumed in the last 28 days?	☐ Beer<br>☐ Wine<br>☐ Spirits/mixed drinks<br>☐ Others
12. How many servings of beer/wine/spirits or mixed drinks did you consume in the last 28 days? (1 serving of beer = approx. 200 ml / 1 glass of wine = approx. 100 ml/ 1 serving of spirits or mixed drinks = approx. 50 ml (of alcohol))	Categories[a]

[a] The different categories were: : Less than once a week / Once or twice a week / 3-4 times a week / 5-6 times a week / Once or twice a day / 3 times or more a day
